# The Zebrafish Cardiac Endothelial Cell—Roles in Development and Regeneration

**DOI:** 10.3390/jcdd8050049

**Published:** 2021-05-01

**Authors:** Vanessa Lowe, Laura Wisniewski, Caroline Pellet-Many

**Affiliations:** 1 Heart Centre, Barts & The London School of Medicine, William Harvey Research Institute, Queen Mary University of London, Charterhouse Square, London EC1M 6BQ, UK; v.lowe@qmul.ac.uk; 2 Centre for Tumour Microenvironment, Barts Cancer Institute, Queen Mary University London, Charterhouse Square, London EC1M 6BQ, UK; l.wisniewski@qmul.ac.uk; 3 Department of Comparative Biomedical Sciences, Royal Veterinary College, 4 Royal College Street, London NW1 0TU, UK

**Keywords:** zebrafish, heart, regeneration, development, coronary vessels, endocardium, lymphatics

## Abstract

In zebrafish, the spatiotemporal development of the vascular system is well described due to its stereotypical nature. However, the cellular and molecular mechanisms orchestrating post-embryonic vascular development, the maintenance of vascular homeostasis, or how coronary vessels integrate into the growing heart are less well studied. In the context of cardiac regeneration, the central cellular mechanism by which the heart regenerates a fully functional myocardium relies on the proliferation of pre-existing cardiomyocytes; the epicardium and the endocardium are also known to play key roles in the regenerative process. Remarkably, revascularisation of the injured tissue occurs within a few hours after cardiac damage, thus generating a vascular network acting as a scaffold for the regenerating myocardium. The activation of the endocardium leads to the secretion of cytokines, further supporting the proliferation of the cardiomyocytes. Although epicardium, endocardium, and myocardium interact with each other to orchestrate heart development and regeneration, in this review, we focus on recent advances in the understanding of the development of the endocardium and the coronary vasculature in zebrafish as well as their pivotal roles in the heart regeneration process.

The zebrafish is a popular small vertebrate model organism used to study embryonic development and, more recently, regeneration, as it is capable of functionally restoring appendages and organs after injury. In this review, we first describe the key stages of endocardium and coronary vessel development and then focus on how these different endothelial cell populations contribute to heart regeneration.

## 1. The Endothelium in Heart Development

In the past 25 years, the zebrafish model has facilitated a wealth of discoveries about angiogenesis and cardiac morphogenesis. It presents several advantages, such as the generation of large sample sets, with a single breeding pair producing ≥200 embryos per mating, their ease of genetic manipulation, and rapid early *ex utero* development. Within 48 h post fertilisation (hpf), the major organs and body plan are completely formed, and the first circulatory loop is established by 24 hpf [1]. Zebrafish embryos are also transparent for the first 24 h of development, after which pigmentation can be suppressed by adding the chemical 1-phenyl 2-thiourea (PTU) to the water or by the breeding of casper, TraNac, or crystal mutant lines, which lack pigmentation throughout adulthood [2,3,4]. 

Because zebrafish embryos are small in size and readily fit within conventional microscopy set ups, they are much easier to investigate than mammalian models. A seminal study by Isogai and colleagues, who used confocal microangiography to observe lumenised vessels, provided a comprehensive description of the first seven days of vascular development, publicly available online: (https://zfish.nichd.nih.gov/Intro%20Page/intro1.html (accessed on 13 January 2021)) [5]. Apart from providing a key resource to the research community, their study also identified that the zebrafish embryonic vasculature is largely stereotypical, i.e., almost all embryos form any given blood vessels with the same pattern at the same timepoint. 

The generation of new transgenic fluorescent reporter lines, advances in microscopy techniques, and the development of morpholino technology, which transiently suppresses mRNA translation, allowed the initial characterisation of molecules central to the development of the vascular system. The use of live in vivo imaging of cardiovascular processes at single-cell resolution enabled the identification of the molecular mechanisms of vessel development in unprecedented detail. Studies describing how signalling pathways involving molecules such as Vascular endothelial growth factor (Vegf), Notch, and C-X-C motif chemokine ligand 12 (Cxcl12)—C-X-C chemokine receptor type 4 (Cxcr4) act together to establish the embryonic vasculature, as well as the discoveries of key angiogenic processes, such as pruning or lumenisation, and the role of perivascular cells have been extensively reviewed elsewhere [6,7,8,9,10,11,12]. The overall advantages, applications, and limitations of the zebrafish model with a particular focus on cardiovascular research were also expertly reviewed by Gut and colleagues [13]. 

The use of morpholinos to simulate the “loss of function” of genes of interest involved in angiogenic processes has been challenged since the observation of Kok and colleagues that a number of genuine loss of function genetic mutant animals failed to phenocopy vascular deformations observed in the corresponding morpholino-injected animals [14]. This caused a significant shift in how morpholinos are used in the laboratory [15] and promoted the adoption of CRISPR/Cas9 and similar gene editing techniques to create mutant allele-harbouring zebrafish lines. Their study also prompted a more nuanced understanding of what underlying biological mechanisms can cause this discrepancy, ranging from within-pathway compensation to cellular stress response and evolutionary divergence of protein function [16,17,18,19]. 

### 1.1. Heart Development in Zebrafish

In vertebrates, the cardiovascular system consists of the heart and all blood and lymphatic vessels in the body. It is the first organ system to become functional in the zebrafish embryo and is crucial to survival past 7 days post fertilisation (dpf). Heart tissue consists of three layers: the myocardium, epicardium, and endocardium. The myocardium is a functional syncytium made up of cardiomyocytes (CM) that contract in response to action potentials induced by pacemaker cells. The endocardium is the inner lining of the heart. It arises from vascular endothelial cells [20] and contributes to the formation of the cardiac valves and part of the coronary vasculature [21,22]. The epicardium is the visceral layer of the pericardium, it is made up of mesothelial cells that protect the heart and stabilise it in its anatomic position. It also supports the coronary vessel network that supplies the heart itself with blood flow. The coronary vasculature arises later during development, from E11 in mice and much later in zebrafish [23].

**Figure 1:**
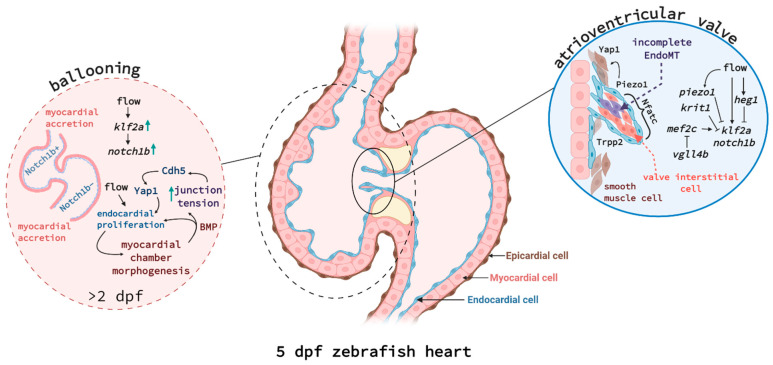
Endocardium-myocardium signalling during ballooning (**left**) and atrioventricular valve formation (**right**). (**Left**) Cell communication between endocardial and myocardial cells is critical for chamber morphogenesis and is also linked to blood flow. Endocardial cells in atrium and ventricle show distinct expression patterns, e.g., ventricular endocardial cells express much higher levels of Notch1b protein. Endocardial cells are proliferating in response to flow and induce myocardial expansion. Myocardial cells do not proliferate during ballooning but rather increase in cell size and by accretion at the poles. Bmp produced by myocardial cells and the increasing junctional tension, mediated by Cadherin-5 (Cdh5) and Yap1, in turn increase endocardial cell proliferation, creating a feed-forward loop. (**Right**) A subset of endocardial cells undergoes incomplete EndoMT to fold into cardiac valves. Tissue tension, mediated by Piezo1 and Trpp2, increases Yap1 expression in some endocardial and surrounding smooth muscle cells to help the elongation of the valve leaflet. Endocardial cells at the tip of the valve are expressing high levels of *klf2a* and *notch1b* in response to flow, but a network of signalling pathways linked to *krit1*, *heg1*, *vgll4b,* and *piezo1* causes downregulation of *klf2a* in endocardial cells closer to the myocardium and cardiac jelly. Interstitial cell differentiation and proliferation is dependent on transcription factor Nfatc. Figure created with Biorender.com.

The zebrafish heart begins forming at 12 hpf when myocardial progenitors migrate from the lateral plate mesoderm to the midline and, by 19 hpf, fuse with endocardial progenitors. Together, they create the cardiac cone that involutes to form the linear heart tube *circa* 24 hpf. Looping occurs from 36 hpf onwards and, by 48 hpf, the distinct curvatures of the atrium and ventricle can be identified. At 72 hpf, a superior valve leaflet has been generated, preventing retrograde blood flow, and by 5 dpf, the heart valve is fully functional, the epicardium fully covers the myocardium, and there is extensive trabeculation (reviewed in [24], see Figure 1). It is important to mention here that zebrafish embryos, unlike mammals or birds, are capable of surviving without circulation for up to 7 dpf [25]. This has enabled research into the contribution of blood flow to cardiovascular development and manipulating blood flow is a key tool in the zebrafish developmental field.

Valve formation, cardiac conduction, ventricular trabeculation, and remodelling all contribute to the maturation of the heart into a fully functional organ and their molecular regulation is the subject of active research. Recently, it was shown that Krit1 *krit1* (also known as Cerebral Cavernous Malformation 1, CCM1) and blood flow-responsive Heart development protein with EGF-like domains 1 (also known as heart of glass, *heg1*) are expressed at sites of high fluid shear stress such as the atrioventricular endocardium. They act there to suppress Kruppel-like factor 2a (*klf2a*) and *notch1b* expression during cushion formation [26]. Additionally, valve formation was demonstrated to depend on Vestigial-like 4 (Vgll4b) sequestration of Myocyte Enhancer Factor 2C (Mef2c), thus suppressing *klf2a* and *notch1b* activation [27]. Two studies have recently identified that the mechanosensory channels Transient receptor potential (Trp) and Piezo1 are expressed by the atrioventricular canal and outflow tract and regulate outflow tract valve morphogenesis [28,29]. Piezo1 negatively regulates *klf2* activity in the endothelial cells forming the valve and positively regulates *yap1* in some endothelial cells and the smooth muscle cells surrounding the endothelium [28], independently of *notch1b* [29]. Yap1 is a Hippo pathway effector and *yap1* mutant embryos displayed valve defects, suggesting that smooth muscle cells contribute to valvulogenesis and require Yap1 to do so [28].

Outflow tract development was described to be reliant on the flow responsive Transforming growth factor β (TGFβ) receptor, Activin A receptor-like type 1 (Acvrl1). Acvrl1 is essential for the accumulation of endocardial cells from aortic arches, the second source of endocardial cells forming the outflow tract [30]. Other groups reported that focal adhesion molecules, especially α5β1 Integrin and Talin1, are key to endocardial cell migration into the developing atrioventricular valve leaflets [31]. Endocardial cells undergo endothelial-to-mesenchymal transition to become interstitial cells involved in valve elongation and function, a process controlled by Nuclear factor of activated T cells 1 (Nfatc1) [32]. These studies highlight the importance of integrating biophysical forces and genetic regulation to understand cardiac development and function. Metabolism also plays a key role in controlling cardiac behaviour: for example, vitamin D promotes cardiomyocyte proliferation during development and in response to injury via ErbB2 [33]. Additionally, trabeculation requires active glycolysis, also under the control of ErbB2 signalling [34].

### 1.2. Endocardial Development and Function

The endocardium is the layer of cardiac endothelial cells that lines the inner surface of the heart tube. Endocardial cells act as blood flow mechanosensors and are a source of angiocrine factors important in the regenerative response after cardiac injury. Endocardial cells also contribute to the overall development of the heart: they form the endocardial cushions, septa, and heart valves; they promote trabeculation, support cardiac conduction, participate in the remodelling of the outflow tract, and are the primary source of coronary vessels (recently reviewed in [35]). 

The exact origin of endocardial cells remains a source of debate and lineage studies suggest that it may differ between model organisms. In zebrafish, all endocardial and myocardial cells originate from the blastula [36]. After gastrulation, a cluster of cells in the anterior lateral plate of the mesoderm expresses endothelial markers such as Etv2 or Tie2, but how and when these cells diverge from the myocardial progenitor cell population remains unclear. In chicks, endocardial and myocardial progenitors are distinct, but in zebrafish embryos, studies using loss of function and overexpression approaches of key transcription factors such as Tal1 or Etv2 have shown that these progenitors are plastic and can switch between the two cell fates [37,38,39]. Genetic lineage tracing studies conducted in mouse embryos over the past ten years support this notion of early progenitor plasticity [40] but importantly also uncovered previously unknown contributions of endocardial (progenitor) cells to the fibroblast, adipocyte, liver vascular cell, haematopoietic cell, and mural cell populations (reviewed in [41]). Furthermore, the contribution of the second heart field to the endocardial pool appears to be of less importance in comparison to mice or quail. Instead, in the zebrafish, the endocardium of the heart chambers is more proliferative (reviewed in [35]). Once specified, zebrafish endocardial cells begin to express typical vascular markers such as Vegfr2, VE-Cadherin, or the endocardial marker Nfatc1 and migrate to the midline, where they form the heart tube together with myocardial cells [42]. This migratory process is dependent on Tal1 as well as the dynamic maintenance of endocardial cell-cell junctional integrity [43,44], while the expression of Nfatc1 itself requires endodermal Hedgehog signalling [45].

After this has occurred, endocardial cells remain on the inner lining but also contribute to different heart tissues, such as the heart valves (via the formation of the endocardial cushions), and developmental processes such as the trabeculation of the myocardium and the generation of the coronary vessels (see Figure 1). To contribute to heart valve formation, a subset of endocardial cells migrates into the cardiac jelly, the extracellular matrix deposited at the sites of valve development by myocardial cells (reviewed in [46]). There, they fold into the valve leaflets, undergoing an incomplete endothelial-to-mesenchymal transition (EndoMT). This is in contrast to mammals where EndoMT gives rise to valve interstitial cells, which form the majority of the valve. A recent study showed that zebrafish also form valve interstitial cells but much later during juvenile development, although the molecular pathways involved appear similar [31].

Endocardial cells also support trabeculation. In mice, this was recently reported to involve endocardial cells sprouting towards the myocardium, thus segmenting the ventricular chamber and encapsulating myocardial cells [47]. In contrast, in zebrafish, trabeculation is largely driven by cardiomyocytes protruding and delaminating into the trabecular layer in response to local tension gradients [48,49]. Additionally, the contractility of the heart affects Klf2 and Notch expression in the ventricular endocardium, thus promoting chamber maturation [50,51]. 

Recent work has further contributed to our understanding of the molecular regulation coordinating endocardial development, which was shown to involve flow responsive Klf2a, Notch, Bone morphogenetic protein (Bmp), and Wnt signalling [52,53,54]. Blood flow acts on the mechanosensitive channel proteins Trpv4 and Trpp2, enabling calcium influx into endocardial cells and inducing Klf2a signalling [55]. Castillo-Robles and colleagues described an early endocardial function for Smarce1, a BRG1/BRM associated factor (BAF) chromatin remodelling complex, via binding to the cis-regulatory regions of the *gata5* gene [56]. In *smarce1* mutants, endocardial cells fail to aggregate in the midline and the atrioventricular boundary is not maintained. Additionally, a study by El-Rass and colleagues found that disrupting platelet-derived receptor alpha (Pdgfra) caused very similar defects in cardiac fusion and endocardial migration to the midline, also affecting atrioventricular chamber development [57]. Another epigenetically-active component, the histone methyl transferase Kmt2d, was shown to control endocardial patterning via Notch signalling [58]. *Kmt2d*-deficient zebrafish are a model of Kabuki Syndrome and mimic some of the congenital disorders observed in patients. Serrano and colleagues showed that Kmt2d, through the suppression of Notch signalling, enables endocardial cells to interact with myocardial cells and thus shape the ventricular lumen. Work from Bornhorst and colleagues further explored the biomechanics regulating overall heart growth during development [59]. The endocardium and myocardium must coordinate to allow functional ballooning of the heart chambers from 30–54 hpf. Endocardial cells actively proliferate during this process, while myocardial cells were shown to largely grow in size or via accretion [60]. Using *nkx2.5*-deficient and *wnt8a* heat-shock overexpressing fish, Bornhorst et al. showed that myocardial size expansion causes mechanical tension in endocardial cell junctions, which is transduced by Cadherin-5, and thus causes the nuclear translocation of Yap1 to induce endocardial cell proliferation ([59], see Figure 1).

### 1.3. Development of the Coronary Vessels Occurs Late during Zebrafish Development

In mice, coronary arteries and most coronary vessels originate from the venous endothelial cells of the sinus venosus, although additional contributions by proepicardial cells and the endocardium have also been reported [61,62,63]. A subset of these cells loses venous identity and acquires a pre-arterial fate before migrating into the myocardium where the plexus is remodelled into veins, arteries, and capillaries. This was shown to be dependent on Vascular endothelial growth factor C (VEGF-C) and Nuclear receptor subfamily 2 group F member 2 (COUP-TFII) activity [23,64,65].

**Figure 2:**
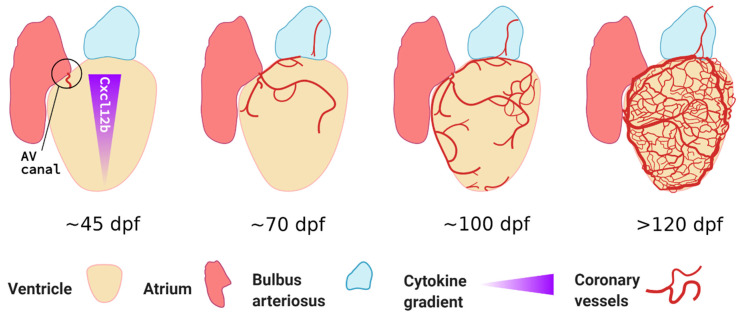
Growth of the coronary vasculature during juvenile and adult life stages of the zebrafish. Coronary vessels emerge late during juvenile development from the atrioventricular canal (AV canal) and continuously grow to cover the ventricular surface; vessel density is correlated to fish age, overall size, and ventricle size. *Cxcl12b* expression is stronger at the base of the ventricle and guides emerging Cxcr4a+ angiogenic sprouts, accordingly, vessel density is higher at the base. Unlike most embryonic vascular beds, the overall patterning is stochastic. While some vessels express arterial markers like *dll4* and *flt1*, venous markers such as *flt4* are absent from the coronary vasculature. Based on confocal microscopy images obtained by Harrison et al. [67]. Figure created with BioRender.com.

It is important to note that, unlike mammals or birds, zebrafish do not form coronary vessels during embryogenesis. This is likely why this developmental process is little studied and in need of more attention by experts in the zebrafish cardiovascular development field. Similarities and differences across different model organisms were recently reviewed by Kapuria et al. [66]. A seminal paper was published by Ching-Ling Lien’s group who first characterised the spatiotemporal development of coronary vessels in zebrafish. Using a combination of fluorescent reporters and chemokine signalling mutants, they demonstrated that coronary vessels only emerge after ~40 dpf, once significant heart maturation has occurred [67]. They showed that this expansion is a gradual process, progressively covering the ventricle from the atrioventricular canal, occurring from ~60 dpf onwards and lasting several weeks (see Figure 2). Unlike most developmental blood vessel growth, coronary vessels appear stochastically. However, the overall vessel density covering the heart surface correlates with age and ventricular size. It is likely that the relatively small size of zebrafish and highly trabeculated myocardium allow sufficient oxygen diffusion before that timepoint. Harrison and colleagues showed that these endothelial cells, rather than originating from the sinus venosus, came from tissue situated between atrium and ventricle, possibly from the endocardium and epicardium [67,68]. They also demonstrated that coronary vessel formation was dependent on Cxcr4a-Cxcl12b signalling as *cxcr4a*-deficient fish or double *cxcl12b/cxcl12a* null fish displayed severely reduced or completely absent coronary vasculature, respectively. Furthermore, they showed that the coronary vessels in zebrafish, unlike other adult vascular beds such as the fin, did not express the venous/lymphatic markers Fms related receptor tyrosine kinase 4 (Flt4) or Prospero Homeobox 1 (Prox1a) [67]. More recently, the group also showed that lymphatic vessels follow the coronary arteries, only sprouting from the bulbus arteriosus from 100 dpf onwards; these lymphatic cells express known markers such as Prox1a, Lymphatic vessel endothelial hyaluronic receptor 1 (Lyve1), and Stabilin 1 (Stab1) [69]. Interestingly, they also demonstrated that lymphatic vessels contribute to cardiac regeneration; this is discussed later in this review. 

What induces the initial outgrowth of the coronary vessels is unknown. One study demonstrated that coronary angiogenesis appears to be independent of Vegfaa, as *vegfaa*-deficient zebrafish embryos, rescued by injection of *vegfaa* mRNA at one-cell-stage, were able to form coronary vessels normally. It is possible that *vegfab* and related genes compensate for this lack of angiogenic cue [70]. Similarly, Vegfaa signalling also appears dispensable for endocardial cell function during early cardiac development, although it contributes to heart tube looping and cardiomyocyte migration to the midline [71]. If and which other angiogenic regulators are important for coronary angiogenesis and how coronary vessel homeostasis is maintained, remain unknown. 

Despite the relative simplicity of the zebrafish heart compared to mammals—i.e., it only has one atrium and one ventricle—many processes involved in heart morphogenesis and homeostasis are conserved across species, making the zebrafish a useful tool for modelling congenital cardiovascular defects. Electrophysiological parameters of the zebrafish heart are also closely aligned with humans—e.g., the electrocardiogram of zebrafish is similar to humans with clearly distinguishable P, QRS, and T waves; ventricular action potentials are comparable; and the heart rate of adult zebrafish (145 bpm) is closer to the human heart rate (60–100 bpm) than that of mice (580–680 bpm) [72,73]. Furthermore, the zebrafish heart expresses orthologues for >95 % of genes implicated in human cardiomyopathy [74].

## 2. The Endothelium in Heart Regeneration

Coronary blood vessel obstruction caused by atherosclerotic plaque rupture or embolism can leave the heart with insufficient blood supply and result in myocardial infarction (MI), more commonly referred to as a heart attack. It is estimated that 1 billion cardiomyocytes are lost during a heart attack in humans [75]. Fibrotic tissue is produced in place of the functional cardiac tissue lost during the MI; this results in cardiac remodelling, hypertrophy, and eventually, heart failure. These devastating complications of heart attacks contribute to 31 % of deaths worldwide, making coronary artery disease the global leading cause of death [76]. Understanding the biological and cellular mechanisms taking place in animal models that can achieve full regeneration of the heart after cardiac injury may identify therapeutic targets to help prevent progression to heart failure or even treat patients. Zebrafish exhibit remarkable regenerative capabilities and can regenerate appendages and organs, including the heart. Full regeneration was shown to be dependent on vessel restoration to the damaged tissue. There are several distinct experimental approaches to induce cardiac regeneration in adult zebrafish which are reviewed elsewhere [77]. Generally, heart injuries are caused by either surgically removing the tip of the ventricle (apical resection), by using a probe cooled in liquid nitrogen (cryoinjury), or genetic ablation approaches which cause extensive and diffuse CM apoptosis.

**Figure 3:**
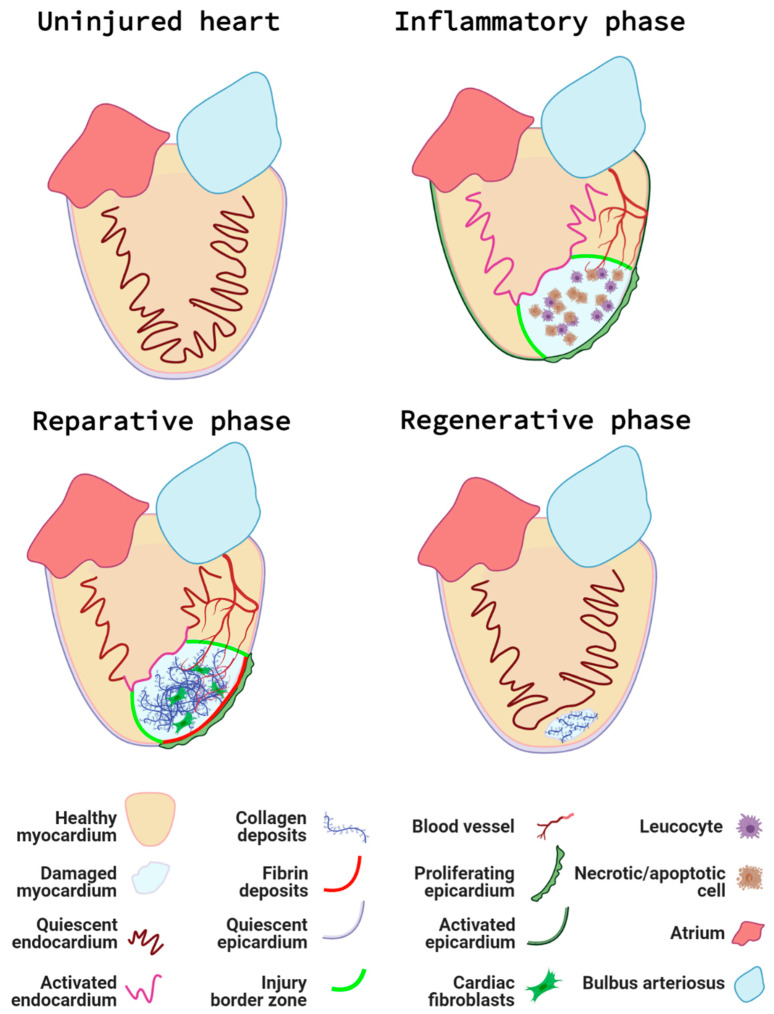
The phases of zebrafish cardiac regeneration. The uninjured heart contains a quiescent endocardium and epicardium, and a healthy myocardial layer. Following cardiac injury, the inflammatory phase takes place. Leucocytes are recruited to the injured area to remove necrotic and apoptotic cells and debris. Additionally, the endocardium and epicardium become activated and the first neovessels infiltrate the injury at the injury border zone (the interface between the injured myocardium and healthy myocardium). During the reparative phase, EPDC, synthetic mural cells and fibroblasts produce an extra-cellular matrix scaffold consisting of a collagen rich core and a fibrin cap. The dynamic signalling and activity of the epicardium and endocardium help coordinate the growth of new blood vessels and support the proliferation of existing cardiomyocytes at the injury border. Next, during the regenerative phase, proliferating cardiomyocytes repopulate the injured area and the fibrotic deposits are gradually removed. Finally, the endocardium and epicardium return to a quiescent state and the final maturation ensures the complete removal of scar tissue, restoration of cardiac function, and synchronicity. Figure created with BioRender.com.

In both the resection and cryoinjury models, a series of cellular events will take place to complete the regeneration of the damaged tissue ([76], see Figure 3).

- First, immediately after the injury and during the next couple of days, an inflammatory response is accompanied by the activation of the endocardium and epicardium. This first step in the regenerative process can be described as the **inflammatory phase**, during which local tissue necrosis and apoptosis result in the recruitment of leukocytes [78];
- Next, a **reparative phase** takes place. Activated endocardial and epicardial cells proliferate and “encase” the wound internally and externally, respectively. Epicardial cells undergo epithelial to mesenchymal transition and give rise to epicardial-derived cells (EPDCs) [79], which, together with the epicardium and fibroblasts, secrete extracellular matrix [80]. As a result, they create a regenerative scaffold that will later support and guide the new cardiomyocytes;
- During the **regenerative phase** per se, pre-existing cardiomyocytes at the border of the injury dedifferentiate, re-express cardiac progenitor markers, and start to proliferate in response to paracrine molecules secreted by neighbouring cells [81,82];
- Finally, a less well-studied **maturation phase** occurs. The regenerated cardiomyocytes re-establish themselves as part of a functional cardiac syncytium and the scar is completely removed [83].

The length of time necessary to complete all phases depends on the model used, with the regeneration following cryoinjury taking longer than the resection or the genetic ablation models because of the need to clear the necrotic tissue and fibrotic scar.

To achieve complete regeneration and restore full cardiac function following MI, the reestablishment of the coronary vessel network is critical, as the delivery of oxygen and nutrients is essential to cater to the high metabolic demands of the heart [84,85]. In adult zebrafish hearts, endothelial cells (EC) represent 37 % of all cardiac cells, nearly equalling the number of cardiomyocytes (CM) (39 %). These numbers are comparable to the relative proportions of CM and EC in mice, nevertheless, the relative ventricular surface area covered by endocardial cells is significantly greater in zebrafish [86]. This highlights the importance of the endothelium and endocardium in the maintenance and regeneration of the myocardium following injury. 

**Figure 4:**
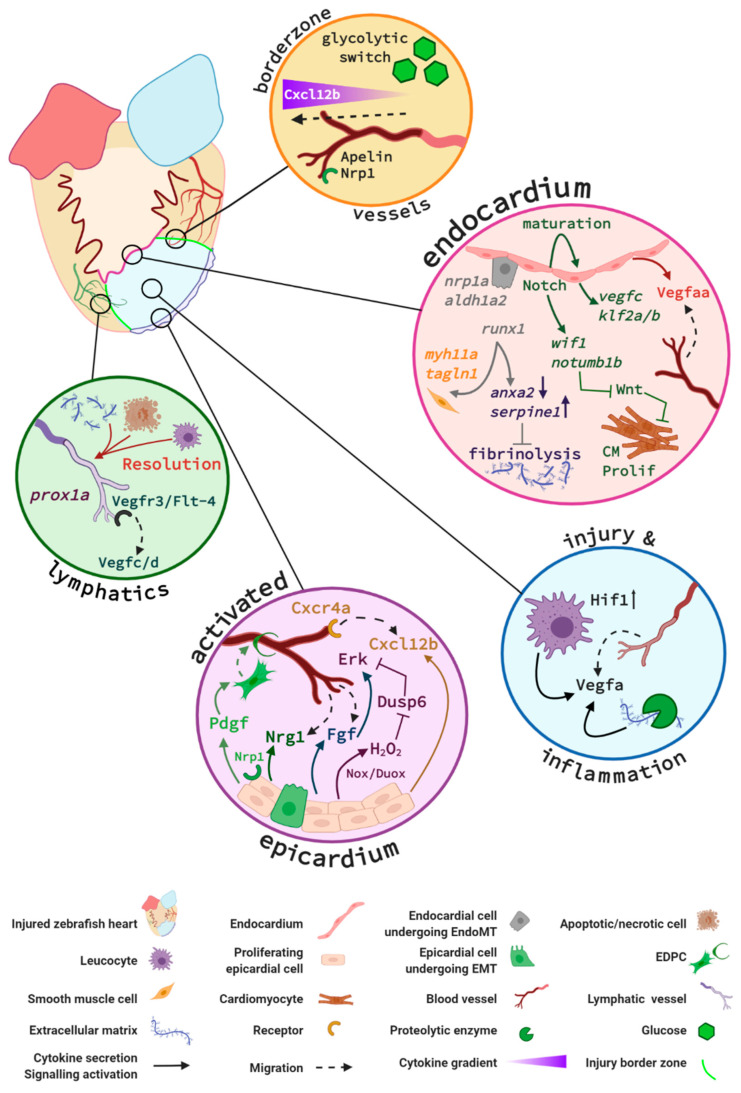
Key signalling pathways in zebrafish endocardium and cardiac vessels during regeneration. Vessels at the injury border (orange circle) undergo a glycolytic switch and use glucose as a primary energy source. Border zone vessels express Apelin and Neuropilin 1 (Nrp1) and respond to Cxcl12b signals that originate at least in part from the activated epicardium. The activated endocardium (pink circle) expresses Notch, which regulates expression of endocardial maturation genes, endothelial differentiation, integrity, and pro-angiogenic genes (e.g., *vegfc*, *klf2a/b*). Additionally, endocardial Notch induces expression of Wnt antagonists *wif1* and *notum1b* that in turn promote cardiomyocyte (CM) proliferation (Prolif). Activated endocardial cells express *nrp1*, *aldh1a2,* and *runx1*. A subpopulation of Runx1-positive cells expresses smooth muscle cell genes *myh11a* and *taglnI*. Runx1 also regulates *anxa2* and *serpine1* and limits fibrinolysis of scar tissue. Within the injury (blue circle), it is hypothesised that recruited macrophages and neutrophils combined with increased Hif1 activity upregulate Vegfa expression. An additional Vegfa source is thought to arise from proteolytic enzyme activity within the injury that releases extracellular matrix (ECM)–bound Vegfa. The activated epicardium (purple circle) secretes Cxcl12b chemokines that bind to the Cxcr4a receptor of endothelial cells in infiltrating vessels. Endothelial cell proliferation is further promoted by epicardial expression of Duox and Nox enzymes that catalyse the generation of hydrogen peroxide (H_2_O_2_). This subsequently inhibits Dusp6 activity in endothelial cells, thus relieving Dusp6 suppression of Erk signalling and enhancing endothelial proliferation. Fgf signalling promotes revascularisation and epicardial activation. Pdgf and Nrp1 promote epicardial activation, a subpopulation of epicardial cells undergo epicardial to mesenchymal transition (EpiMT). These epicardial-derived cells (EPDCs) become fibroblasts secreting ECM and perivascular cells. Nrg1 expression by EPDCs further promotes angiogenesis. *Prox1a*-expressing lymphatic vessels are detected in the injury and respond to Vegfc/d via Vegfr3. Inflammatory cells, necrotic and apoptotic cells, and extracellular matrix debris are removed via the lymphatic vessels (green circle) to aid the regenerative process. Figure generated with BioRender.com.

In the following part of this review, we discuss the cellular mechanisms and signalling pathways that have been reported to play essential roles in the restoration of the coronary vascular network and endocardial response in the regenerating zebrafish heart (summarised in Figure 4).

### 2.1. Early Cellular Mechanisms Contributing to Coronary Revascularisation Following Cardiac Injury

#### 2.1.1. Inflammation

Following myocardial infarction, chronic inflammation exacerbates fibrotic deposits and subsequent cardiac remodelling [87], which led to the hypothesis that inflammation is an inhibitor of cardiac repair. Nevertheless, when the acute inflammatory response is experimentally abrogated following cardiac injury in the zebrafish, heart regeneration is impeded via mechanisms that are still incompletely understood, but are at least partially due to the disruption of neo-vessel formation [78,88,89,90]. Neo-vessel infiltration is observed in the damaged cardiac tissue as early as 15 h post-cryoinjury (hpci) [70], which is concomitant with immune cell infiltration during the initial acute inflammatory phase. Since leucocytes are known to be potent mediators of angiogenesis, it could be hypothesised that inflammation stimulates this initial vascular recruitment via the production of Vegf and other angiogenic factors by the infiltrating neutrophils and macrophages [91,92,93,94]. Inflammation is a prerequisite for the revascularisation of the injured zebrafish heart [78,90] and likely one of several biological responses that directly and indirectly promote angiogenesis in the regenerating zebrafish heart; however, the underlying molecular mechanisms remain grossly undercharacterised. It is essential to investigate the interplay between infiltrating leucocytes, the endocardium, and coronary vasculature to better understand the pro-angiogenic effects elicited by inflammation in zebrafish heart regeneration.

#### 2.1.2. Endothelial Cell Migration

The importance of restoring the vascular network to the damaged myocardium is supported by the observation of infiltrating vessels within the injured area as early as 15 hpci and the reestablishment of a vascular network by 2 days post-cryoinjury (dpci) [70]. Nevertheless, at this timepoint, the myocardium at the site of injury is largely necrotic and does not require any supply in oxygen or nutrients. Marin-Juez et al. recently hypothesised that this early revascularisation leads to the formation of a vascular network acting as a scaffold and supporting cardiomyocyte proliferation via a process they termed “coronary-endocardial anchoring” [95]. Proliferating cardiomyocytes are found to interact closely with the neovessels, indicating that they act as a structural framework. Although it is likely that paracrine factors are secreted by the neovessels to attract and support cardiomyocytes replenishment of the injured area, the exact molecular mechanisms that support this interaction are not yet elucidated. Additionally, they demonstrated that new coronary vessels first sprouted superficially within the activated epicardium and then invaded the ventricular tissue in depth, toward the activated endocardium. The molecular mechanisms promoting this biphasic neovascularisation are described later in this review. Their study also strongly suggests that the main source of the regenerated coronary vasculature are pre-existing coronary vessels rather than endocardial cells [95,96]. However, specific endocardial lineage-tracing experiments need to be completed to indisputably preclude their contribution to the regenerating coronary vasculature.

#### 2.1.3. Metabolic Modulation

Endothelial cells in the cryoinjury border zone display an acute glycolytic switch. Expression analysis of the oxidative metabolism regulator gene, peroxisome proliferator-activated receptor gamma coactivator 1a (*ppargc1a*) was reduced in actively sprouting and proliferating coronary ECs sorted from 96 hpci hearts [97]. Moreover, a mutant zebrafish line lacking *ppargc1a* displayed increased coronary vessels density within the injured area, indicating its limiting role on EC proliferation [95]. In contrast, the glycolysis promoting gene *apelin*, which is expressed in activated endothelial cells after injury [97], was upregulated and associated with increased EC proliferation [95]. In addition to its impact on metabolic modulation, Apelin also guides the lead sprouting endothelial cells as its expression can be readily identified in the extending sprouts at early time points following cardiac injury [95]. Thus, this study provides evidence that ECs acutely switch their metabolic programme during the early stages of regeneration, likely to tolerate the hypoxic environment of the injured area. Indeed, *apelin* and other glycolysis-promoting genes are regulated by *hif* expression in ECs in the ischemic heart [98,99].

### 2.2. Pro-Angiogenic Molecules in Zebrafish Cardiac Revascularisation and Regeneration

Since a variety of cell types are involved in cardiac regeneration, several cytokines and growth factors have been identified as key molecular regulators, with many of them being conserved between zebrafish and mammalian species. Below, we list those that have been shown to play a role in the angiogenic response to cardiac injury and describe their role in orchestrating cellular repair (see Table 1 and Table 2).

#### 2.2.1. Vascular Endothelial Growth Factor

Vascular endothelial growth factor (VEGF) is a well-characterised and potent pro-angiogenic peptide essential for the development of the coronary vasculature [100,101]. It comes as no surprise that Vegf is also a critical regulator of zebrafish neovascularisation following cardiac injury. Vegfaa signalling was shown to mediate early revascularisation using a transgenic zebrafish expressing a dominant negative *vegfaa* under the control of a temperature-inducible heat shock 70-like (*hsp70l*) promoter [70]. The authors reported that *vegfaa*^−/−^ mutant fish displayed a reduced vessel density at the infarct zone, which was accompanied by a decrease in cardiomyocyte proliferation, permanent scar formation, and a failure to fully regenerate. Interestingly, *vegfaa* gene expression increases at 24 hpci, hours after the initial angiogenic sprouting; thus, Vegfaa bioavailability may originate from proteolytic release of local extracellular matrix-bound Vegfaa [102] or could also be secreted by the recruited leucocytes [78]. Indeed, *vegfaa* expression was reduced in 1-day post-amputation (dpa) zebrafish hearts that were treated with an anti-inflammatory glucocorticoid used to dampen the early inflammatory response, thus impairing revascularisation, and resulting in an incomplete regeneration [78]. Another recent study by Marin-Juez and colleagues demonstrated that endocardial Vegfaa signalling regulates intra-ventricular coronary vessel invasion into the regenerating myocardium, towards the activated endocardium of the regenerating heart. In particular, their study showed that, in *flt1*^−/−^ mutant zebrafish with enhanced Vegfaa signalling, the length and density of intra-coronary vessels in the injured area increased, and more cardiomyocytes were found to proliferate and associate with vessels [95]. Additional evidence for the role of Vegfaa as an important regulator of CM-endothelium crosstalk was demonstrated by Karra et al. who found that the overexpression of *vegfaa* drives CM hyperplasia and activates regenerative programmes in the endocardium (and epicardium) following cardiac injury [103]. 

Therefore, the key role for Vegf in the angiogenesis/revascularisation processes previously described in other models was also validated in the zebrafish model of heart regeneration. As the number of specific zebrafish mutant lines increases, we are likely to find out more about the tight spatial and temporal control of Vegf and its role in heart repair.

#### 2.2.2. Neuregulin1

Neuregulin1 (Nrg1) is a transmembrane protein which, after proteolytic cleavage by metalloproteinases or A disintegrin and metalloproteinases (ADAM), becomes a signal peptide that binds to ErbB2 and ErbB4 receptor tyrosine kinases. Neuregulin signalling is essential for zebrafish cardiogenesis [104,105] and a potent mitogen for several cardiac cell types [106]. In zebrafish, stimulation of *nrg1* activity was found to be sufficient to induce the heart regeneration programme and angiogenesis in uninjured hearts [107]. Gemberling and colleagues also reported that Nrg1 promotes zebrafish heart regeneration by stimulating coronary revascularisation as well as CM proliferation in amputated zebrafish hearts. Additionally, their study suggests that one source of Nrg1 originates from the epicardial-derived perivascular cell population, which therefore may play a supporting role for vessel growth following apical resection [107]. T_reg_ cells recruited to the cardiac injury site also secrete Nrg1 and this was accompanied by a strong upregulation of its receptors (*erbb2*, *erbb4a* and *erbb4b*) by CM [108]. The exact source and mechanisms by which Nrg1 achieves its pro-angiogenic properties need to be further elucidated and cell-specific zebrafish lines are essential tools to achieve that purpose.

#### 2.2.3. Notch

Notch signalling is a well-characterised regulator of endothelial tip cell differentiation during mammalian and zebrafish developmental angiogenesis [109,110,111]. In zebrafish, Notch is upregulated in the epicardium and the endocardium after cardiac resection. Suppression of Notch signalling profoundly impairs cardiac regeneration and induces scar formation at the amputation site [112]. Nevertheless, Münch et al. concluded that Notch signalling is dispensable for endocardial activation and for the revascularisation of the regenerating heart. This is because coronary vessels repopulate the regenerating tissue in the absence of Notch [112], despite the concurrent aberrant regeneration of the myocardium [96]. More recently, the same group further characterised the specific role for endocardial Notch signalling and identified Wnt antagonists *notum1b* and *wif1* as Notch target genes that dampen Wnt signalling and ultimately boost CM proliferation [113]. Additionally, when Münch and colleagues investigated the role of endocardial Notch using pharmacological inhibition and genetic overactivation strategies, they found that Notch signalling was important for endocardial maturation. Comparing the transcriptomes of Notch-inhibited versus control cryoinjured ventricles, they demonstrated that Notch regulates genes involved in the regulation of angiogenesis (*vegfc*, *id1*, *efnb2a*, *egr1*), endothelial integrity (*cldn5b*, *heg1*), and endothelial cell differentiation (*klf2a*, *klf2b*, *aqp1a.1*). Interestingly, they also identified plasminogen activator inhibitor 1 (*serpine1*), a fibrinolysis inhibitor, as being upregulated upon Notch inhibition [112]. 

These studies demonstrate a central role for Notch in modulating CM proliferation and scar resorption via endocardial maturation. Secretion of endothelial paracrine factors, such as Wnt antagonists, to the myocardium is also key to successful cardiac repair [112,113].

#### 2.2.4. Cxcl12—Cxcr4 signalling

The EC chemokine receptor 4a (Cxcr4a) is essential for coronary vasculature guidance in zebrafish juvenile development. *Cxcr4a* mutant zebrafish do not develop coronary vasculature and those few that survive to adulthood exhibit severely impaired cardiac regenerative capacity [67]. Marín-Juez and colleagues made the interesting observation that *hif1*^−/−^ zebrafish hearts displayed reduced epicardial *cxcl12b* expression after cryoinjury, along with a decrease in superficial coronary cell proliferation. Conversely, increasing Hif1a activity with Dimethyloxallyl Glycine (DMOG) induced endothelial proliferation, which led to the conclusion that hypoxia triggers epicardial *cxcl12b* expression via Hif1a [95]. They also noted that *cxcr4a* mutant hearts were largely devoid of new superficial coronaries at 7 dpci whilst control hearts were fully covered by this time point. This led them to postulate that the activated epicardium, secreting Cxcl12b, guides superficial coronary ECs expressing Cxcr4a to modulate revascularization [95]. As described earlier, these superficial coronary vessels will then be attracted by endocardial-derived Vegfaa, to invade the myocardium and form intraventricular coronary vessels. Taken together, this study provides extensive insight into the multi-tissue signalling pathways that govern revascularisation in the zebrafish heart regeneration model.

### 2.3. The Epicardium and Endocardium Support Neovascularisation and Vessel Maturation

The epicardium is a quiescent single mesothelial cell layer encasing the heart. It is activated upon cardiac injury. Epicardial cells start proliferating and a subpopulation displays morphological changes, migrates into the damaged tissue via a process called epicardial-to-mesenchymal transition (EpiMT), and thus participates in the regenerative process [79]. The activated epicardium secretes paracrine factors to stimulate cardiomyocyte proliferation, fibroblast recruitment, and vessel growth [120,121,122]. In the following paragraphs, we discuss the molecules secreted by the activated epicardium that are likely to be important paracrine factors supporting coronary vessel regeneration as well as endocardial activation during zebrafish heart regeneration (see Table 1 and Table 2). 

#### 2.3.1. Fibroblast Growth Factor

The supporting role of the activated epicardium in the revascularisation of the regenerating myocardium was initially demonstrated by Lepilina et al. [79]. Fibroblast growth factor (FGF) was already known to be a pro-angiogenic mitogen with a key role in wound healing angiogenesis [123]. In their study, Lepilina and colleagues demonstrated that Fgf was essential for the EpiMT and neovascularisation of the injured tissue, both crucial events in the regeneration process. Indeed, in the absence of Fgf signalling, activated epicardial cells expressing *tbx18* failed to migrate into the regenerating myocardium, coronary neovascularisation failed to occur, and a permanent scar was deposited [79]. Recent evidence also describes a potential role for Fgf signalling via the Extracellular Signal-Regulated Kinase (Erk) downstream pathway in the regenerating heart [124]. Erk phosphorylation is known to be a key event common to several signalling pathways modulated by angiogenic growth factors [125]; therefore, Erk phosphorylation observed in endothelial cells after cardiac injury may contribute to heart regeneration via modulating revascularization [124].

Other cell types also mediate Fgf signalling in the zebrafish regenerating heart. Zebrafish treated with an anti-inflammatory glucocorticoid, thereby suppressing the early immune cells infiltration to the wound, had a significant reduction in cardiac regeneration capability. Huang and colleagues observed a decreased revascularisation of the injured tissue in parallel with a reduction of mRNA expression of fibroblast growth factor receptor 1 (*fgfr1*). Therefore, suppressing the early inflammatory response severely hinders the zebrafish heart regenerative capacity, at least in part via inhibiting Fgf signalling and its function in mediating revascularisation [78].

#### 2.3.2. Platelet Derived Growth Factor

The platelet derived growth factor (Pdgf) signalling pathway plays a critical pro-angiogenic role in zebrafish heart regeneration. An upregulation of *pdgfrβ* and other EpiMT markers such as *snail* and *twist1b* are detected in the epicardium after apical resection [114]. In their study, Kim et al. hypothesised that epicardial cells undergoing EpiMT during zebrafish heart regeneration are likely to contribute to the establishment of mural cells and fibroblasts surrounding the coronary vessels. This was later confirmed by González-Rosa and colleagues, using epicardial specific transgenic lines [114,115]. Pharmacological inhibition of Pdgfrβ in amputated zebrafish hearts impaired epicardial activation and subsequently hampered the pro-angiogenic paracrine signals derived from the activated epicardium [114]. Thus, Pdgf indirectly stimulates revascularisation via activation of the epicardium during zebrafish heart regeneration.

#### 2.3.3. Neuropilins

The transmembrane glycoprotein neuropilins (NRP1 and NRP2) are co-receptors required for the development of the nervous and vascular system [126]. Nrps morphant fish develop vascular defects and revealed synergistic interactions with Vegf to mediate developmental angiogenesis [127]. Additionally, as mentioned above, Neuropilin ligands such as Vegf, Pdgf, and Fgf were reported as important regulators of zebrafish cardiac injury-induced epicardial activation and revascularisation [70,79,114,115,116,117,118,119,120,121,122,123,124,125,126,127,128,129,130], thus suggesting a role for Nrp1 signalling in zebrafish heart regeneration. Following cryoinjury, zebrafish *nrps* are strongly upregulated in the activated epicardium [116], matching expression patterns of previously reported co-receptor pathways [79,114]. Nrp1 is also expressed by the endocardium and the coronary vessels, both in haemostasis and during the regenerative process. In particular, activated endocardial cells detaching from the underlying myocardium and migrating into the injured cardiac tissue strongly overexpress Nrp1 [116]. Because NRP1 is known to promote epithelial-to-mesenchymal transition in other settings [131,132], it was hypothesised that, by analogy, Nrp1 might be involved in the modulation of endocardial-to-mesenchymal transition (EndoMT) in zebrafish heart regeneration. In *nrp1a* mutant fish, the impaired epicardial activation and a marked reduction in the number of new coronary vessels both likely contribute to the hindered scar resolution and delayed overall cardiac regeneration observed [116]. 

Because Nrps are able to bind several co-receptors to mediate multiple ligands’ signalling pathways in different cell populations, but also because each Nrp isoform has two ohnologues in the zebrafish, compensatory mechanisms are likely to complicate the interpretation of the specific Nrp1 and Nrp2 roles in the zebrafish model. Further studies are needed to fully assess which parts of this complex signalling network contribute to cardiac regeneration.

#### 2.3.4. Runx1

Runx1 is a master transcription factor that plays a key role in modulating the proliferation of several cell types, both during development and adulthood. *Runx1* loss of function affects multiple cell types within the regenerating heart but, in wild-type controls, was strikingly overexpressed by the epicardium and the endocardium following cryoinjury [133]. The scar composition of *runx1* mutants differs from wild-type control fish and its resorption is significantly faster. Interestingly, sorted Runx1-positive endothelial/endocardial cell populations were very heterogenous, with subsets of cells expressing collagens, EndoMT genes, and/or smooth muscle genes after injury. The *runx1* mutation led to the loss of an endocardial cluster expressing smooth muscle cell *myh11a* and *taglnI*, potentially explaining the difference in scar composition [133]. Additionally, the endocardium of *runx1* mutant fish also showed deregulated genes involved in fibrinolysis, including an increase in plasminogen receptor annexin A2 (*anxa2*) and a decrease in *serpine1* expression in comparison to wild-type hearts. This led the authors to further suggest that, in addition to the differential scar composition, increased fibrinolysis in the *runx1* mutant underlies the reduced amount of fibrin observed in the wound [133].

#### 2.3.5. NOX/Duox

Following zebrafish cardiac injury, the avascular damaged tissue becomes hypoxic and initiates an oxidative stress response via the production of reactive oxygen species (ROS) such as hydrogen peroxide (H_2_O_2_). ROS, in turn, directly interact with key downstream molecules to trigger signalling in a broad range of cellular processes [134]. In zebrafish, the epicardial generation of H_2_O_2_ catalysed by the NADPH-oxidase (Nox) enzymes, Duox and Nox2, promotes heart regeneration by a derepressing mechanism, whereby H_2_O_2_ weakens the activity of the redox-sensitive phosphatase Dusp6, thus increasing Erk1/2 phosphorylation in response to growth factor signalling [117,118]. Pharmacological inhibition of Duox and Nox significantly reduced cardiomyocyte proliferation, Flk1-positive coronary vessel density and prevented regeneration in amputated zebrafish hearts [118]. A subsequent study supported these observations and confirmed *dusp6* expression in endothelial cells and the epicardium of regenerating zebrafish hearts [119]. Neovascularisation was increased in *dusp6* mutant zebrafish hearts and this aligned with enhanced Erk phosphorylation and accelerated cardiac regeneration [119]. Although the cell-specific contributions towards revascularisation and heart regeneration by endothelial and epicardial Nox2/Duox signalling are yet to be fully characterised, these reports provide clear evidence for ROS in promoting coronary vessel growth in regenerating zebrafish hearts, likely—at least in part—by epicardial activation and production of H_2_O_2_.

#### 2.3.6. Aldh1a2

The retinoic acid (RA)-synthesising enzyme Aldh1a2 is rapidly expressed by the entire endocardium following resection of the apex of the heart before becoming localised to the injury zone the day after. The expression of *aldh1a2* coincides in time and space with endocardial cell morphological changes, which appear rounded and detach from the underlying myocardium, likely reflecting increased permeability [135]. *Aldh1a2* expression was specifically induced in endothelial cells of the endocardium as no expression was noted in the coronary vessels or the aorta’s endothelia. Additionally, robust upregulation was also observed in the epicardium and, similarly to the endocardial expression, was first triggered as an organ-wide response before becoming restricted to the injury site [79,135]. In their study, Kikuchi and colleagues further characterised the permissive role for endocardial and epicardial-derived Aldh1a2 in supporting CM proliferation during the regeneration process [135].

### 2.4. The Lymphatic Vasculature in Zebrafish Heart Regeneration

Mammalian studies demonstrated that lymphatic vessels respond to myocardial infarction by increasing in length and density; the extent of this response is associated with an improved cardiac outcome [136,137,138,139]. Regulators of zebrafish lymphangiogenesis such as Vegfc, Vegfr3, and Cxcl12b [140,141] have been previously reported to be upregulated after zebrafish cardiac injury [67,69,116,136,138,142]. However, like the regulation of developmental lymphangiogenesis, studies focused on the contribution of lymphatic vessels to overall zebrafish regeneration processes and heart regeneration remain limited. The development, functions, and disease models related to cardiac lymphatics in the zebrafish heart have recently been extensively discussed by Feng and colleagues [143]. Below, we outline some of the key findings of how lymphatics contribute to zebrafish heart regeneration.

An extensive cardiac lymphatic vascular network develops in mammalian embryonic hearts [136]. In contrast, the zebrafish ventricular lymphatic vessels, much like the coronary vessels, only develop in late juvenile/early adulthood stages in zebrafish, at approximately 3 months post fertilisation [144,145]. The zebrafish ventricular lymphatic vessel network is located in the subepicardial layer; it includes a dominant lymphatic vessel that spans the length of the ventricle from outflow tract to the base to apex and sprouts to form a lymphatic network, in conjunction with isolated lymphatic colonies detected sporadically throughout the ventricle [69,145]. Similar to mammals, abnormal or absent cardiac lymphatic network formation is associated with pathological defects, chronic inflammation, and fibrosis [139,146,147].

Apical resection generates a mild lymphangiogenic response, whereas neolymphangiogenesis is robustly stimulated globally in cryoinjured zebrafish ventricles, implicating differential requirements for lymphatic vessels when cardiac tissue is damaged in cryoinjury versus the removal of tissue in the resection model [69]. Cryoinjury results in increased lymphatic endothelial cell (LEC) proliferation, enlarged and extensively branched lymphatic vessels in the injured area, and elevated *vegfc* expression until 42 dpci [69,146]. Prox1a expressing neolymphatic vessels can be detected in the cryoinjured area at 40 hpci. These *de novo* lymphatic vessels then sprout and form connections, providing the primary neolymphatic vessel cell source during the first 14 days after cardiac injury. Lymphatic vessels derived from the outflow tract take 1–3 weeks to reach the injury area after cryoinjury and contribute to a smaller proportion of lymphatic vasculature in the injured region [145]. Leading edge LECs express *flt4* and sprout in response to Vegfc during lymphatic vessel development in the zebrafish; conversely, *flt4*^−/−^, *vegfc*^+/−^ zebrafish hearts were unable to form neolymphatic vasculature in the cryoinjured area, resulting in increased scar tissue deposition and abolition of the regenerative capacity in cryoinjured zebrafish hearts [145]. A subset of *vegfc*^−/−^, *vegfd*^−/−^ double mutant zebrafish hearts failed to regenerate after cryoinjury and displayed a persistent scar [146]. In accordance with these observations, transgenic fish that overexpress soluble Flt4, thus inhibiting Vegfc activity, also exhibit impaired scar resolution that correlates with reduced lymphatic vasculature [69]. 

Extensive inflammation, necrosis, and apoptosis of cardiac cells is observed following cardiac insult. The inflammation and cellular debris are resolved and subsequent scar resorbed in regenerating zebrafish hearts; thus, biological mechanisms orchestrating these clearance mechanisms must also influence heart regeneration [90]. A study by Vivien and colleagues elucidated a lymphatic role in apoptotic cell clearance during the acute inflammatory phase at 1 dpci [146]. *Vegfc*^−/−^ and *vegfd*^−/−^ null zebrafish that fail to develop lymphatic vessels in the heart presented an accumulation of TUNEL staining in the injured tissue, indicating that dead cells remain in the injury site. These findings agree with a study by Harrison and colleagues that detected immune cells in the lymphatic vasculature of cryoinjured hearts but not in uninjured heart lymphatic vessels [69]. Furthermore, inhibition of Vegfc signalling resulted in persistent detection of neutrophils in the wound at 14 dpci [69]. 

Re-establishment of the lymphatic vessels in the injured area of zebrafish hearts likely governs the clearance process during cardiac regeneration. Given that new blood vessels infiltrate the injured area within hours of initial cardiac insult and that LECs migrate alongside coronary vessels as a guidance [69,145], it is possible that neolymphatic vessels use the neovasculature to access the injury site and mediate clearance of inflammatory cells in response to cardiac damage. Aberrant lymphatic vasculature does not affect epicardial activation, blood vessel growth, or cardiomyocyte proliferation, suggesting that the resultant scar in lymphatic-compromised regenerating zebrafish hearts is due to chronic inflammation and subsequent pro-fibrotic activity [145,146]. Further work is needed to fully characterise ventricular drainage and inflammatory resolution in regenerating zebrafish hearts.

## 3. Summary

The zebrafish is a popular model organism that has allowed the observation of developmental processes in vivo in real time, greatly expanding our knowledge of the cardiovascular system. Increasingly sophisticated tools enable us to gain a better molecular and cellular understanding of how blood flow, contractility, and myocardial, epicardial, and endocardial cells all act together to generate a functional cardiovascular system. 

The importance of the revascularisation of the injured heart has only been elucidated recently. Indirect or direct impairment of the signalling molecules or cell types involved in revascularisation results in incomplete regeneration. Neovessel growth takes place very rapidly after cardiac damage and is a prerequisite for the subsequent regenerative processes. Multiple tissues and signalling pathways are key to achieving a mature vascular network, highlighting the importance revascularisation has on subsequent regeneration. Although several studies have identified angiogenic candidates that govern heart regeneration, it is still an undercharacterised field that warrants further investigation. The body of evidence gathered from studies thus far comes from different models of myocardial infarction. It is plausible that differential mechanisms of revascularisation between zebrafish models and regenerating mammalian models take place. To achieve translational benefits, it is important to tease out which pathways are conserved and able to stimulate revascularisation in adult mammalian hearts. Revascularisation is key to regeneration regardless of the organ, but it is only one of several processes that we need to fully understand and re-initiate if we are to successfully mend the broken heart.

## Figures and Tables

**Table 1:** Angiogenic gene regulation in the regenerating zebrafish heart. Clear rows indicate genes upregulated after cardiac injury, grey shaded rows indicate genes that are downregulated. CM—cardiomyocyte, EC—endothelial cell, ECM—extracellular matrix, EpiMT—epithelial-to-mesenchymal transition, *dn*—dominant negative, *Tg*—transgenic.

Gene	Source	Role	Transgenic Phenotype If Known	Reference
*vegfaa*	Infiltrating leucocytes ECM release?	Pro-angiogenic peptide Early revascularisation	*vegfaa^−/−^*: reduced infarct vessel density.	[70,78,91,92,93,94,102]
	Endocardium	Intraventricular revascularisation, CM proliferation & migration	*flt1^−/−^, βact2:BS-vegfaa*: increased infarct vessel density and increased CM proliferation (due to increased *vegfaa* activity).	[95,103]
*ppargc1a*	Leading edge ECs	Oxidative metabolism regulator	*ppargc1a^−/−^*: increased infarct vessel density	[95,97]
*apelin*	Leading edge ECs	Glycolysis regulator ECs guidance and proliferation	Not determined	[95,97,98]
*hif*	Not determined	Regulate hypoxia & glycolytic genes. Epicardial activation	*hif1^−/−^*: reduced epicardial *cxcl12b* expression and coronary vessel growth	[98,99]
*cxcr4a*	ECs	Coronary vessel proliferation and maturation	*cxcr4^−/−^*: reduced coronary vessel growth.	[95]
*cxcl12b*	Epicardium	Guides *cxcr4* positive ECs to modulate revascularisation	Not determined	[95]
*fgf*	Epicardium Infiltrating leucocytes	EpiMT and revascularisation	*Tg(dn-fgfr1)*: reduced infarct vessel density	[78,79]
*pdgf*	Epicardium	EpiMT, neovessels maturation	Not determined	[114,115]
*nrp1a*	Epicardium Endocardium Coronary ECs	Epicardial activation, revascularisation	*nrp1a^−/−^*: reduced infarct vessel density and reduced epicardial activation	[116]
*nox/duox*	Epicardium	Revascularisation and CM proliferation	Not determined	[117,118]
*dusp6*	Endothelium Epicardium	neovascularisation	*dusp6^−/−^*: increased vessel density	[119]

**Table 2:** Angiocrine signals mediated by the regenerating zebrafish heart endothelial/endocardial cell populations. Clear rows indicate genes upregulated after cardiac injury, grey shaded rows indicate genes that are downregulated. CM—cardiomyocyte, EC—endothelial cell, EPDCs—Epicardial-derived cells, ECM—extracellular matrix, EndoMT—endothelial-to-mesenchymal transition, *dn*—dominant negative, *Tg*—transgenic.

Gene	Source	Role	Transgenic Phenotype If Known	Reference
*nrg1*	EPDCs	Coronary revascularisation, CM proliferation	*Tg(cmlc2:CreER; β-act2:BSNrg1):* Increased revascularisation and CM proliferation	[107]
*notch*	Endocardium Epicardium	CM proliferation and scar resorption via endocardial maturation	*Tg(UAS:NCID; ET33-mi60a)*: endocardial maturation *Tg(dn-MAML)^EC^:* reduced CM proliferation and increased fibrosis	[112,113]
*runx1*	Endocardium Epicardium	EndoMT Fibrosis	*runx1^−/−^*: accelerated scar resorption and decreased fibrosis, increased CM proliferation	[133]
*aldh1a2*	Endocardium Epicardium	CM proliferation	*Tg(Hsp70:dn-zrar)*: decreased CM proliferation	[135]

## Data Availability

Data sharing not applicable.
